# Adjustment of the MIND diet tool for discriminating Greek patients with dementia: A confirmatory factor analysis

**DOI:** 10.3389/fneur.2022.811314

**Published:** 2022-09-07

**Authors:** Emilia Vassilopoulou, Lemonica Koumbi, Calliope Karastogiannidou, Panagiotis Marios Sotiriadis, Pop Claudia Felicia, Magda Tsolaki

**Affiliations:** ^1^Department of Nutritional Sciences and Dietetics, International Hellenic University, Thessaloniki, Greece; ^2^Department of Nursing “Iuliu Hatieganu”, Department of Medicine and Pharmacy, University of Medicine and Pharmacy, Cluj-Napoca, Romania; ^3^1st Department of Neurology, Aristotle University of Thessaloniki, AHEPA University Hospital, Thessaloniki, Greece; ^4^Greek Alzheimer Association and Related Disorders, Thessaloniki, Greece

**Keywords:** MIND diet, Greek population, aging, confirmatory factor analysis, nutrition, cross-sectional study, cognition, dementia

## Abstract

**Background:**

The MIND diet, a hybrid of the Mediterranean and DASH diets, has been shown to reduce cognitive decline and dementia occurrence.

**Aim:**

In the current cross-sectional study the effect of the MIND diet in elderly Greek individuals, assessed for cognitive decline, was investigated. Confirmatory factor analysis (CFA) evaluated the MIND diet score's factor structure in relation to the ability to distinguish the Greek elderly population diagnosed with or without dementia.

**Methods:**

One hundred fifteen participants recently diagnosed with dementia and 52 cognitively healthy controls, after proper neuropsychological testing by neurologists, were included. To ensure the variance-covariance of matrix for the CFA, a second reference group of 36 participants who self-reported as healthy in terms of cognitive status from the general Greek population, was included. Demographic, anthropometric characteristics, emotional status, cognitive function, and dementia diagnosis were recorded. A prediction model investigated the MIND diet's components to separate the study participants according to their cognitive health. CFA was used to examine if the structure of the MIND diet tool scale was a proper model fit or if a different model more appropriately fit our sample data.

**Results and discussion:**

The CFA conducted, suggested that the 9 components MIND diet score supported our sample data better than the original 15-item MIND diet.

**Conclusion:**

The MIND diets' components must be considered in relevance to the dietary habits and cultural background of the respective population studied. Future studies should evaluate prospectively the effect of MIND-9 on preventing the onset of dementia in Greek adults.

## Introduction

The prevalence of dementia has increased dramatically as life expectancy has extended (1). Cognitive decline, which is the hallmark of dementia, is common in the elderly: nearly 50 million people are living with dementia worldwide, and this number is expected to increase to over 80 million by 2030 (2).

The most common cause of dementia is Alzheimer's disease (AD), a neurodegenerative disease characterized by progressive cognitive impairment. The neuropathology of AD involves complex molecular pathways, including the accumulation of the beta-amyloid peptide (Aβ) in the brain, cytoskeletal changes due to the hyperphosphorylation of microtubule-associated Tau protein in neurons, oxidative stress and neuroiflammation (3). The neuroinflammatory mechanisms have been associated with immunosenescence and intestinal dysbiosis (4). Despite many years of intensive research, no effective treatment is available, nor proven preventative interventions for the cognitive loss in dementia and AD (2).

Cognitive decline is not an inevitable consequence of aging, and a number of protective modifiable lifestyle factors have been identified to counteract it. Diet is a lifestyle factor that can be changed, and several dietary patterns have been associated in numerous studies (5–11) with a protective effect on cognitive decline and dementia development. Increased risk of dementia has been associated with consumption of food high in saturated fat and simple carbohydrates, termed the “Western” diet, while diets high in mono- and poly-unsaturated fats, vegetables, and lean proteins are linked with reduced risk (12). The Mediterranean diet resembles such a protective diet, as it includes a high intake of olive oil, pulses and vegetables, moderate consumption of fish and poultry, a low intake of dairy products, and a limited consumption of red meat and sugar (13). As it is rich in antioxidants and anti-inflammatory substances, the Mediterranean-style diet has been proposed to confer protection against metabolic syndrome, obesity, cardiovascular, and neurodegenerative diseases (14–16).

Morris and his team formulated the Mediterranean-DASH diet intervention for neurodegenerative delay (MIND) diet which is a hybrid of the Mediterranean diet and the dietary approaches to stop hypertension (DASH) diet (10). An increasing number of studies has demonstrated that adherence to the MIND diet strongly associates with lower AD risk and with a slower decline in brain function (1, 7, 10, 12, 17–19). The MIND diet is characterized by the high consumption of 10 plant-based foods, such as berries and green leafy vegetables as well as by the limited intake of unhealthy animal foods and saturated fats (7). Those 10 beneficial dietary components have been associated with the prevention of cognitive decline and dementia, via their antioxidative, anti-inflammatory, and neuroprotective properties (10, 11, 20–22). The MIND diet may benefit patients with AD by reducing potentially harmful Aβ proteins, via alterations of the gut microbiome composition and the effect of the antioxidants and vitamins (23, 24). In addition, the MIND diet limits saturated and trans fats which have been shown to halt Aβ clearance in the brain and their intake has been associated with increased Aβ deposition and higher AD risk (21, 22, 25).

The risk of developing dementia has been linked to anxiety and depression possibly due to the reduced function of neurotransmission pathways during cognitive impairment (26). Susceptibility to anxiety and depression is known to be influenced by the microbiome-gut-brain axis, which is ultimately shaped by diet (16). During dementia's development, depressive and anxiety symptoms that overlap with incremental cognitive impairment can lead to the deterioration of dietary habits affecting diet adherence and hence may result in reverse casuality (22).

In this study, confirmatory factor analysis was used to test the original the MIND diet scale's factor structure (construct validity) in relation to the ability to distinguish the Greek elderly population diagnosed with or without dementia.

## Materials and methods

### Study population

The study sample consisted of three groups:

Group A included 115 elderly individuals with cognitive decline and diagnosis of dementia (dementia group).

Group B included 52 participants diagnosed with no cognitive decline (healthy controls).

Group C included 36 participants originating from the general Greek population, at the same age range as Group B, who self-reported as healthy, but were not assessed for their cognitive status by neurologists (second control group).

Group A and Group B participants have been referred for the first time for cognitive evaluation to the Hellenic Association for Alzheimer's Disease and Related Disorders in Thessaloniki, Greece from January 2021 to April 2022. Standardized neuropsychological testing for the diagnosis of cognitive decline was conducted by neurologists, according to the International Classification of Diseases, 11th Revision (ICD11) (27). For the purposes of this study, the diagnosis of the neuropsychological testing of cognitive decline and dementia was retrieved from the medical records of the participants after their evaluation, and they were grouped in Group A or B according to diagnosis on their cognitive status and the Mini mental state exam (MMSE) (28).

Group C was included in the study to increase the number of participants and to ensure the variance-covariance matrix of the CFA (29).

All participants provided their written consent to participate in the study, after being informed about the protocol and procedures of the study. The study was approved by the Bioethics committee of Alzheimer Hellas (64/09-01-2021) and was conducted between January 2021 and April 2022.

### Anthropometric measurements

Participants' anthropometric measurements were collected by one trained investigator on the morning of the interview, after fasting for at least 8 h. Height was measured to the nearest 0.1 cm, using a commercial stadiometer (Leicester Height Measure, Invicta Plastics Ltd, Oadby, UK) with the participants barefoot, their shoulders in a relaxed position, their arms hanging freely and their heads in the Frankfort horizontal plane. The participants were weighed barefoot and in light clothing to the nearest 0.1 kg, using a TANITA RD-545 (“RD-545-Connected smart scale Tanita Official Store,” n.d.). Body mass index (BMI) was calculated from the current weight and height [weight (kg) by height squared (m^2^)] and classified accordingly into the following BMI categories: underweight (<18.5), healthy weight (18.5–24.9), overweight (25–29.9) and obese (>30) (27).

### Dietary habits

A certified dietitian interviewed the participants regarding their dietary habits with the tool derived based on the MIND scoring matrix published by Morris and colleagues (10) (Supplementary Table 1). The interview were done directly to the participants, in most of the cases with the presence of their caregivers, who assisted them to respond with accuracy.

In the original scoring of the MIND diet, 15 dietary parameters were considered, of them 10 were so-called as brain healthy food groups (green leafy vegetables, other vegetables, nuts, berries, olive oil, whole grains, fish not fried, poultry not fried, beans, and wine) and five as brain unhealthy food groups (red meat and meat products, butter and margarine, cheese, pastries and sweets, and fast fried food) (10). Scoring ranged from 0 to 15 (minimal to maximal adherence to the MIND diet). A value of 0, 0.5, or 1 was assigned for intake of each dietary component based on predefined cut-offs. For olive oil, fish, whole grains, berries, green leafy vegetables, other vegetables, nuts, beans, and poultry, a value of 1 was assigned to high intake. For butter, margarine, cheese, red meat and products, fast fried foods, and pastries and sweets a value of 1 was assigned to low intake. For wine, a value of 1 was assigned to moderate intake. The total MIND diet score was the sum of the 15 component scores. Adherence to the MIND diet was considered low for scores between 2.5 and 6.5, moderate between 6.5 and 8.5 and high from 8.5 to 15.

### Evaluation of negative emotions (depression, anxiety and stress)

The depression anxiety stress scale (DASS-21), validated for the Greek population (30) was used to evaluate the emotional status of the participants in terms of depression, anxiety and stress. The questionnaire included 3 scales, each consisting of 14 items, rated on a 4-point Likert scale, with higher scores indicating more severe symptoms.

### Statistical analysis

Statistical analysis was conducted using the SPSS v.24 statistical package and p-level was set at 0.05. The values were expressed as mean ± standard deviation (±SD) and the difference between the participants' characteristics were screened with the independent samples *t*-test.

Confirmatory factor analyses (CFA) were used to examine the construct validity of the scales of the original MIND diet tool for the Greek population. The efficiency of the total score of the MIND scale was assessed in the differentiation between the patient and control groups.

CFA was performed using the AMOS program, version 22. Using AMOS 22, the χ^2^/df, TLI, CFI and RMSEA Goodness-of-Fit (GFIs) were examined to test whether the model fitted the data well (29). A good model fit is implied when χ^2^/df <5 and ideally non-significant, TLI > 0.90, CFI, > 0.90 and RMSEA <0.08 (31). We were particularly focused on the TLI, which is relatively unaffected by sample size. Modifications indices were examined to identify the observed variables that deteriorated the GFI of the model. A variable was excluded from the model when it decreased its GFIs and scale alpha reliability and concurrently either did not contribute to discriminate between people with dementia and healthy controls or discriminated them to the opposite direction. Based on this methodology, three models consisted of 15, 11, and 9 variables, respectively, were identified and presented below. The total scale score was computed for the best model. Likewise, based on the acceptable alpha's Cronbach, the scale score of DASS-21 was computed. To investigate the association of DASS-21 with MIND, DASS-21 was regressed on MIND controlling for gender, age and BMI. Furthermore, MMSE was regressed on MIND, DASS-21, gender, age and BMI to investigate the convergent and divergent validity of MIND scale before using it to discriminate participants with dementia from healthy controls.

The dementia group alongside with the healthy control group formed a dichotomous variable (0 = no dementia, 1 = yes dementia). The latter was used as Dependent Variable (DV) in two logistic regression models, the first included gender, age, BMI, DASS-21 and MIND as Independent Variables (IVs), while the second model included the four aforementioned IVs plus MMSE. Finally, to investigate whether each diet component can discriminate participants from healthy controls similar to the total MIND score, this binary DV was regressed on each diet component across 15 separate logistic regressions, controlling for gender, age, BMI and DASS-21 in each analysis.

## Results

### Descriptive statistics

The characteristics of the study participants are presented in Table 1. Group A consisted of 115 individuals with dementia (71.3% women), out of which 34 were diagnosed with early onset dementia. The mean age of Group A was 72.57 (±8.08) years, slightly higher than the mean age of the 52 healthy controls (75% women) of Group B (70.17 ± 4.61 years) and the self-reported as healthy 36 individuals (50% women) of Group C (68.92 ± 6.24 years). The mean BMI of Group B (26.72 ± 3.15) was lower than the BMI of Group A 28.02 (± 4.45) (*p* = 0.01) and, Group C (28.67 ± 4.82) (*p* = 0.02).

**Table 1:** Comparative presentation of the demographic, anthropometric, clinical characteristics and dietary intake of the study participants.

**Parameter**	**Group A (*n* = 115)**	**Group B (*n* = 52)**	***p*-value^a^**	**Group C (*n* = 36)**	***p*-value^b^**
Gender (male)	33 (28%)	13 (25%)	0.383	18 (50%)	0.015
Age	72.57 ± 8.08	70.17 ± 4.61	0.048	68.92+6.24	0.308
Bmi	28.20 ± 4.45	26.72 ± 3.15	0.015	28.67 ± 4.82	0.024
Mmse	25.32 ± 5.15	29.02 ± 0.91	0.000	N/A	N/A
Dass_score	26.38 ± 21.40	16.35 ± 11.96	0.000	20.11 ± 19.63	0.267
**Food item**
Green leafy vegetables	0.47 ± 0.33	0.62 ± 0.37	0.016	0.44 ± 0.31	0.021
Other vegetables	0.68 ± 0.29	0.42 ± 0.40	0.000	0.42 ± 0.35	0.937
Berries	0.07 ± 0.18	0.25 ± 0.40	0.002	0.26 ± 0.42	0.876
Nuts	0.37 ± 0.36	0.70 ± 0.30	<0.001	0.47 ± 0.37	<0.001
Whole grains	0.36 ± 0.32	0.40 ± 0.31	0.421	0.32 ± 0.42	0.307
Cheese	0.44 ± 0.34	0.26 ± 0.29	0.001	0.38 ± 0.35	0.105
Beans	0.51 ± 0.24	0.44 ± 0.21	0.076	0.39 ± 0.24	0.290
Olive oil	0.98 ± 0.11	1.00 ± 0.00	0.103	0.96 ± 0.18	0.183
Butter and Margarine	0.74 ± 0.33	0.91 ± 0.26	<0.001	0.61 ± 0.42	<0.001
Poultry not fried	0.48 ± 0.26	0.52 ± 0.34	0.496	0.51 ± 0.35	0.943
Red Meat and Meat Products	0.86 ± 0.23	0.89 ± 0.30	0.479	0.85 ± 0.31	0.484
Fish not fried	0.47 ± 0.32	0.88 ± 0.24	<0.001	0.67 ± 0.38	0.005
Fast fried food	0.87 ± 0.26	0.97 ± 0.15	0.002	0.78 ± 0.30	0.001
Pastries and Sweets	0.47 ± 0.34	0.73 ± 0.40	<0.001	0.85 ± 0.29	0.117
Wine	0.23 ± 0.32	0.38 ± 0.30	0.005	0.33 ± 0.40	0.594
Mind score	7.43 ± 1.88	9.38 ± 1.48	<0.001	8.25 ± 1.69	0.002

Unless otherwise described, data are presented as mean ± SD.

Different superscript letters (a, b) indicate comparison among Groups A and B or Groups B and C, respectively, at a significance level of p ≤ 0.05.

Group A: individuals with dementia; Group B: healthy controls; Group C: self-reported as healthy individuals.

DASS-21 score was significantly higher in Group A (26.38 ± 21.40) than in Group B (16.35 ± 11.96) (*p* < 0.001), while it did not differ significantly from groups B and C (*p* = 0.26).

Regarding their dietary habits, overall moderate adherence to the MIND diet was recorded in Group A (7.43 ± 1.88) and Group C (8.25 ± 1.69), while adherence in Group B was in the high range (9.38 ± 1.48) (*p* < 0.001). Statistically significant differences were accounted for green leafy vegetables, nuts, berries, whole grains, fish not fried, wine, butter and margarine, pastries, sweets and fast fried food (*p* ≤ 0.05) among Group B and Group A.

In addition, Group B consumed significantly higher amounts of green leafy vegetables, nuts, butter/margarine, fish and fast fry food in relevance to Group C (*p* ≤ 0.05) (Table 1). There was no variation in olive oil among study participants, as almost all (98%) used it as the main cooking oil.

### Prediction model

**Figure 1:**
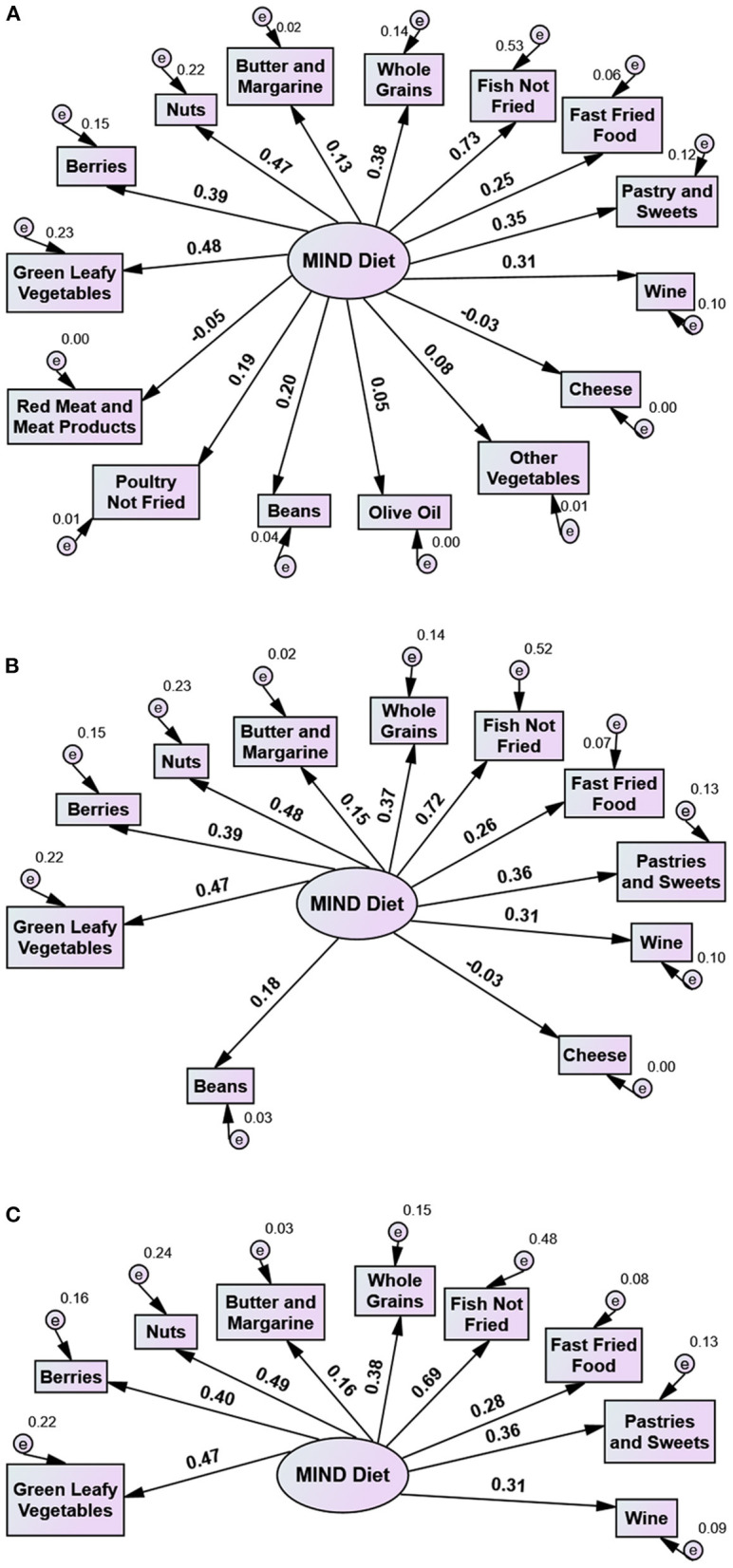
Use of the MIND Diet tool in a Greek adult population with dementia (patient group) and without dementia (control group): Three 1 factor models with 15, 11, and 9 variables. Numbers on arrows indicate factor loadings (standardized beta weights). **(A)** Model 1. All diet components of MIND Diet tool: Green Leafy Vegetables, Other Vegetables, Berries, Nuts, Olive Oil, Whole Grains, Beans, Fish Not Fried, Poultry Not Fried, Butter and Margarine, Cheese, Red Meat and Meat Products, Fast Fried Foods, Pastries and Sweets, Wine). χ^2^ = 158.703, *p* < 0.001, df = 90, χ^2^/df = 1.763, CFI = 0.660, TLI = 0.604, RMSEA = 0.061, **(B)** Model 2. MIND Diet tool minus Other Vegetables, Berries, Nuts, Whole Grains, Beans, Fish Not Fried, Butter and Margarine, Cheese, Fast Fried Foods, Pastries and Sweets, Wine. χ^2^ = 53.481, *p* = 0.155, df = 44, χ^2^/df = 1.215, CFI = 0.932, TLI = 0.915, RMSEA = 0.033, **(C)** Model 3. MIND-9 Diet tool i.e. MIND Diet tool minus Other Vegetables, Berries, Nuts, Whole Grains, Fish Not Fried, Butter and Margarine, Fast Fried Foods, Pastries and Sweets, Wine). χ^2^ = 35.581, *p* = 0.125, df = 27, χ^2^/df = 1.318, CFI = 0.937, TLI = 0.916, RMSEA = 0.040.

A prediction model was created to investigate the MIND diet's components to separate the study participants according to their cognitive health. Initially, the 15 dietary parameters of the original MIND diet tool constituted one factor termed MIND in the first model of the present CFAs. All residuals were assumed to be uncorrelated (Figure 1) and maximum likelihood estimation was used.

CFA using a model with all 15 diet components as suggested by Morris et al. (7) did not fit the data well (results shown in Figure 1) while some diet components failed to properly separate the participants from healthy controls. Results from modification indices suggested that four diet components, namely olive oil, poultry not fried, other vegetables, red meat and meat products, did not differentiate statistically among Group A and Groups B and C. Therefore they had a negative effect on the GFIs, while their loadings were also very low (Model 1 in Figure 1A) and contributed negatively to the reliability of the MIND scale. Consequently, when they were excluded, the scale alpha reliability increased from 0.55 to 0.63. Accordingly, these four diet components were excluded from the model and a new model consisted of the remaining eleven diet components was examined (Model 2 in Figure 1B).

While Model 2 fit the data well, the scale alpha reliability including the 11 diet components was still low. Further investigation of the diet components revealed that beans and cheese- also without statistical difference in consumption among Groups A, B, and C contributed negatively to the reliability of the scale, while the loading of cheese was zero (Figure 1B). Beans did not discriminate participants with dementia from healthy controls, while cheese contributed to an opposite direction. Exclusion of these two diet components from the MIND scale increased Cronbach's alpha from 0.63 to 0.67.

Accordingly, cheese and beans were excluded from Model 2 and a new model consisted of the nine remaining variables was examined (Model 3). As shown in Figure 1C, this model fit the data well. We further computed a score for MIND-9 scale, which was the sum of scores across the nine diet components. The overall mean MIND-9 score in all study participants was 4.61 (± 1.56) (median 4.5, minimum 0.5–maximum 8). The mean MIND-9 score in Group B (5.84 ± 1.11) was significantly higher than in Group A (4.05 ± 1.41) and Group C (4.74 ± 1.35) (*p* < 0.001).

Results from fifteen separate logistic regression analyses for each one of all fifteen MIND diet components, using each diet component as IV and existence of dementia as DV, controlling for gender, age, BMI and DASS-21 are summarized in Table 2 with the Odds Ratios (OR) being presented long with their 95% Confidence intervals (CIs). These findings imply that each diet component of MIND-9, except of whole grains and green leafy vegetables, significantly discriminated participants with dementia from healthy controls.

**Table 2:** Odds ratios (OR) and Confidence intervals 95% (CI) of all MIND diet components from nine separate logistic regression analyses controlling for gender, age, BMI and DASS-21 affecting patient and healthy control groups separation.

**Diet components of MIND**	**OR**	**95% CI**	
		**Lower**	**Upper**
Green leafy vegetables	0.36	0.13	1.05
Other vegetables	7.82***	2.57	23.80
Berries	0.14**	0.04	0.51
Nuts	0.06***	0.02	0.20
Olive oil	0.99	0.000	–
Butter and margarine	0.11**	0.03	0.49
Cheese	15.84***	3.95	63.55
Whole grains	1.04	0.34	3.23
Fish not fried	0.01***	<0.01	0.07
Beans	3.49	0.77	15.86
Poultry not fried	0.66	0.21	2.10
Red meat and meat products	0.66	0.16	2.74
Fast fried food	0.06*	<0.01	0.69
Pastries and sweets	0.14***	0.04	0.39
Wine	0.21*	0.07	0.67

*** p <0.001; ** p <0.005; * p <0.05.

DASS-21 had a high alpha reliability (alpha = 0.91) allowing us to calculate the total DASS-21 scale score. Then DASS-21 was regressed on gender, BMI and MIND-9. Results showed that following a significant adjustment of gender (implying that females had higher scores on DASS-21), still MIND-9 accounted for significant variance of DASS-21 (Table 3). The association of MIND-9 and DASS-21 was negative.

**Table 3:** Standard regression analyses of DASS21 and MMSE on MIND-9, gender, age and BMI.

	**Dependent variable**			
	**DASS21**		**MMSE**	
	**β**	***r***	**β**	***r***
Gender	0.24***	0.25***	0.03	0.06
Age	0.03	0.02	−0.29***	−0.35***
BMI	−0.07	−0.03	−0.02	−0.04
MIND-9	−0.17*	−0.18**	0.24***	0.32***
DASS21	–	–	−0.11	0.14

β, standardized beta *** p <0.001, ** p <0.01, * p <0.05.

Results from the next standard regression analysis of MMSE on MIND-9, gender, age, BMI and DASS-21 revealed that after adjusting on age effects (implying that older people had lower scores on MMSE), MIND-9 accounted for significant variance of MMSE.

Considering dementia absence as DV and gender, age, BMI, DASS-21 and MIND-9 as IVs, our results shown in Table 3 demonstrate that, after significant adjustment of DASS-21, MIND-9 accounted for unique variance of absence of dementia. Odds ratios imply that with a one-unit increase in MIND-9 score the odds of being a person with dementia decrease about 3 times.

The same logistic regression was computed with the inclusion of MMSE into the equation as an additional IV. Results shown in Table 4 (Model 2) shows that after adjusting for the significant effects of MMSE, MIND-9 still discriminated healthy controls from participants with dementia.

**Table 4:** Odds ratios (OR) and 95% confidence intervals (CI) from logistic regression analysis of absence of dementia as a function of MIND-9, gender, age, BMI DASS-21 and MMSE.

	**Dementia yes-no**			
	**Model 1**		**Model 2**	
	**OR**	**CI 95%**	**OR**	**CI 95%**
Gender	0.42	0.15–1.15	0.37	0.12–1.14
Age	1.01	0.94-1.07	0.97	0.90–1.04
BMI	1.11	0.99–1.24	1.12	0.98–1.27
DASS-21	1.04**	1.01–1.07	1.03*	1.01–1.06
Mind-9	0.36***	0.25-0.52	0.43***	0.29-0.63
MMSE	–	–	0.73**	0.57-0.93

***p <0.001, **p <0.01, *p <0.05.

The results from second logistic regression (Model 2) revealed that MMSE accounted for unique significance variance of dementia. Still, MIND-9 accounted for the additional significant variance of dementia than the significant variance of dementia accounted for due to MMSE.

## Discussion

In this cross-sectional study we investigated for the first time the construct validity of the MIND diet tool and its respective items among Greek individuals with and without diagnosis of dementia. The dementia group had a significantly lower overall adherence to the MIND diet, compared to the healthy group. The confirmatory factor analysis (CFA) revealed that the score of the nine of the fifteen items that compose the original MIND diet, could discriminate the participants with dementia from the healthy control group. Therefore, we propose herein that the MIND-9 score for the evaluation of the Greek elderly population. Dementia occurrence was positively correlated with the age, BMI, and emotional burden according to DASS-21.

The impact of the MIND diet on cognitive decline has been investigated by relatively few studies since it was first described in 2015 (7, 10, 12, 17, 18). The MIND diet was formulated by Morris and his team, in a cohort prospective study, the RUSH Memory and Aging Project (MAP) in Chicago, USA (10). Studies since then have used a 144-item modified Harvard semi-quantitative FFQ to determine the MIND diet scores, and have documented that adherence to the MIND diet improves cognitive decline, mood and disability, in both subjects experiencing healthy aging and those with dementia (7, 10, 12, 17). In the understudy population herein, we aimed to capture the MIND diet components in the Greek population; the overall score of nine of the fifteen MIND diet items, lower than 4.5 out of 9, efficiently discriminated participants with dementia from healthy participants.

The MIND diet is based on the components of the Mediterranean and DASH diets, and emphasizes the intake of plant-based foods, fish, nuts and berries, components known to have antioxidative, anti-inflammatory, and neuroprotective activities, while the consumption of animal and saturated fat foods is limited. Foods and nutrients included in the MIND diet have been shown to prevent cognitive decline (10, 32). Our study groups had similar consumption of six diet components, specifically increased intake of olive oil and other vegetables, moderate intake of cheese, poultry and beans and low intake of red meat, in line to the traditional Mediterranean diet (7, 11, 21). Therefore, these components had a negative effect on the GFI of the questionnaire.

Every food component of the MIND-9 except of whole grains and green leafy vegetables significantly discriminated dementia participants from healthy controls. Future research with larger sample of Greek participants may show the beneficial effect of green leafy vegetables on cognitive function, as they are a rich source of vitamin E, folate, β-carotene, phylloquinone, and lutein, which account for their neuroprotective role (8, 32, 33). A high intake of berries has been shown to slow the decline in cognitive function; berries are a rich source of anthocyanidins, which have been shown to be transported through the blood-brain barrier to the brain areas of memory and learning processing (9). A number of studies have shown negative correlation between moderate fish consumption and occurrence of AD (34). Fish is high in omega-3 fatty acids (n-3 FAs), which exert protective properties against AD by their ability to reduce oxidative stress, to lower insulin and cholesterol levels and to decrease the risk for cardiovascular disease (34). Nuts have been associated with improved cognition; they contain high levels of polyunsaturated fats and antioxidant polyphenols that can promote neuronal function through additive effects, and regulate cardiovascular risk factors (8).

Following a significant adjustment of gender, implying that females had higher scores on DASS-21, our results showed that MIND-9 accounted for significant variance of DASS-21 and confirmed that people with less depressive and anxiety symptoms have a higher adherence to the MIND diet. Longitudinal studies have shown that depression and anxiety are risk factors for the development of dementia (26). A recent prospective study in the USA demonstrated that mild anxiety symptoms alone can predict dementia risk (35). Female sex significantly correlates with anxiety and dementia development (35–37). Raparelli et al. (2020) showed, by multivariate analysis, that low adherence to the Mediterranean diet was associated with male sex and perceived stress, but not with disease progression (37). Further studies are required to explore the role of gender, specific personality traits and the emotional status in the development and progression of dementia, and in the application of therapeutic dietary interventions. In addition, our participants with dementia had a significantly higher BMI than the healthy control group. Previous studies also demonstrated a significant positive association of BMI and the likehood to develop dementia (38).

Overall, in the present study, after significant adjustments of DASS-21, gender and MMSE scores, MIND-9 still accounted for unique variance of absence of dementia. The significant negative correlation between the MIND-9 and DASS-21 psychometric measures supports the divergent validity of MIND-9, in agreement with previous studies on the 9 food items (7, 10, 12, 17, 18). Our results suggest that MIND diets' components must be considered in relevance to the dietary habits and cultural background of the understudy population. For instance, in Greece and probably in other areas of the Mediterranean basin, where olive oil is the main cooking oil (19) it's beneficial effect on dementia was not observable. Moreover, the overall contribution of olive oil in the diet should be considered. Thus, the last must be considered as a significant limitation of the MIND diet tool; our results show that it cannot capture effectively either the dietary habits of the understudy and it's adherence to the guidelines for a healthy diet (39) or the more prominent components of the Greek diet that may relate to dementia. Thus, in order to capture the food items that could be significant for dementia, a food frequency questionnaire relevant for the Greek diet should have been used in parallel to the MIND diet tool. In addition, our study did not include the education attainment throughout life, which according to the cognitive reserve theory can protect significantly against cognitive decline and is a strong predictor of cognition (40). Another possible limitation of our study is that it is based on the recall of the frequency of consumption of various food components and people with dementia might not be able to recall past information accurately, increasing this way the probability of the recall bias, especially when the caregiver was not present during the interview or was not responsible for the food preparation.

## Conclusion

Our findings support the construction validity of the MIND diet tool after reducing its initial components from 15 to 9 in relation to the ability to distinguish the Greek population with or without dementia. Future studies should aim to evaluate prospectively the effect of MIND-9 on preventing the onset of dementia in Greek adults and probably other Mediterranean populations with similar eating habits. Our study is indicative, that the effectiveness of the MIND diet in preventing dementia, depends on the consumption of specific functional diet components, also relevant to the eating pattern of the understudy population.

## Data availability statement

The raw data supporting the conclusions of this article will be made available by the authors, without undue reservation.

## Ethics statement

The studies involving human participants were reviewed and approved by Bioethics committee of Alzheimer Hellas (64/09-01-2021). The patients/participants provided their written informed consent to participate in this study.

## Author contributions

Conception: EV,MT, and PCF; data collection: EV and PMS; data interpretation: EV, CK, and PCF; data analysis: CK; drafted the article: EV, CK, PCF, and LK; revised and approved final article: all authors.

## Conflict of interest

Author MT was employed by Greek Alzheimer Association and Related Disorders. The remaining authors declare that the research was conducted in the absence of any commercial or financial relationships that could be construed as a potential conflict of interest.

## Publisher's note

All claims expressed in this article are solely those of the authors and do not necessarily represent those of their affiliated organizations, or those of the publisher, the editors and the reviewers. Any product that may be evaluated in this article, or claim that may be made by its manufacturer, is not guaranteed or endorsed by the publisher.
