# Supplementary material for: Adjustment of the MIND diet tool for discriminating Greek patients with dementia: A confirmatory factor analysis
Source: Front Neurol. 2022 Sep 7;13:811314. doi: 10.3389/fneur.2022.811314 (PMC9516637; doi:10.3389/fneur.2022.811314)
Supplement: Supplementary file 1 [file Table_1.docx]

**APPENDIX**

**Dietary component, servings and maximum score for the MIND diet score.**

| **MIND Diet Score** | |
| --- | --- |
| **MIND components** | **Max Score** |
| **Whole Grains**  ≥ 3/d | **1** |
| **Green Leafy**  ≥ 6wk | **1** |
| **Other vegetabels**  ≥  **1/d** | **1** |
| **Berries**  ≥ 2/wk | **1** |
| **Red Meats and products <4/wk** | **1** |
| **Fish**  ≥ 1/wk | **1** |
| **Poultry**  ≥ 2/wk | **1** |
| **Beans > 3/wk** | **1** |
| **Nuts**  ≥ 5/wk | **1** |
| **Fast/fried food < 1/wk** | **1** |
| **Olive oil primary oil** | **1** |
| **Butter, margarine < 1T/d** | **1** |
| **Cheese <1/wk** | **1** |
| **Alcohos/wine 1/d** | **1** |
| **Total MIND Score** | **15** |

**d= day**

**wk= week**
